# Clinical study of eculizumab with or with not tacrolimus in the treatment of generalized myasthenia gravis

**DOI:** 10.3389/fimmu.2026.1906981

**Published:** 2026-10-07

**Authors:** Bin Yang, Jing Wang, Xiuli Zhao, Chuya Jing, Jian Yin, Liyan Qiao

**Affiliations:** 1 School of Clinical Medicine, Tsinghua University School of Clinical Medicine, Beijing, China; 2 Department of Neurology, Tsinghua University Yuquan Hospital, Beijing, China; 3 Department of Neurology, Beijing Hospital, Beijing, China

**Keywords:** eculizumab, generalized myasthenia gravis, MG-ADL, minimal symptom expression, QMG, tacrolimus

## Abstract

**Introduction:**

Eculizumab, an antibody that inhibits the complement system, and the immunosuppressant Tacrolimus are both authorized for treating patients with generalized myasthenia gravis (GMG). This study seeks to evaluate and compare the clinical effectiveness of Eculizumab with or with not Tacrolimus in the treatment of generalized myasthenia gravis.

**Methods:**

A retrospective study was performed involving 95 patients with GMG treated with Eculizumab at three hospitals in China. Among them, 80 patients were included in the analysis: 57 received Eculizumab alone, while 23 were treated with a combination of Eculizumab and Tacrolimus. The main outcome assessed was the percentage of GMG patients who reached Minimal Symptom Expression (MSE). Secondary outcomes included changes in MG-Activities of Daily Living (MG-ADL) scale, Quantitative Myasthenia Gravis (QMG) score, CH50 concentration, and doses of pyridostigmine and prednisolone. Additionally, the safety profiles of both treatment groups were examined.

**Results:**

In the main outcome, a higher proportion of patients reached MSE status when treated with a combination of Eculizumab and Tacrolimus compared to Eculizumab alone (Eculizumab: 29 patients, 50.9% vs Eculizumab and Tacrolimus: 18 patients, 78.3%; p<0.05). Regarding secondary outcomes, both treatment groups showed significant improvements in clinical symptoms, as indicated by notable reductions in QMG and MG-ADL scores, along with a significant reduction in prednisolone dosage. Furthermore, analysis of secondary outcome parameters revealed no significant differences between the two groups (p>0.05). The decrease in complement CH50 concentration is accompanied by a decrease in QMG and MG-ADL scores, indicating that Eculizumab treats GMG through the mechanism of complement system. In terms of security, hyperuricemia was more commonly associated with the combination therapy of Eculizumab and Tacrolimus than with Eculizumab alone.

**Conclusion:**

Our research indicates that Eculizumab can effectively reduce disability in GMG patients by inhibiting complement system activation under real-world clinical conditions in China. We observed a correlative link between Eculizumab and Tacrolimus combination treatment and an increased probability of achieving MSE status, in comparison with Eculizumab monotherapy. However, combination therapy is associated with a higher risk of elevated serum uric acid among GMG patients, which necessitates regular close monitoring of renal function.

## Introduction

Generalized myasthenia gravis (GMG) is an uncommon autoimmune disorder (1). It is characterized by intense muscle fatigue and weakness during physical activity, causing challenges with movement, speech, swallowing, eye movements, and breathing (2, 3). Approximately 18% of individuals with GMG may undergo a potentially life-threatening myasthenic crisis, which involves respiratory failure and necessitates mechanical ventilation (4). Minimal manifestations status (MMS) is the goal of clinical comprehensive treatment of GMG, but it is limited by nonstandard qualitative evaluation and prominent physician subjective bias, and poor repeatability of multicenter statistical comparison (5). On the contrary, minimum symptom expression (MSE) is a completely quantitative and objective patient reporting endpoint, defined as MG-ADL score 0 or 1, and has become a simple, interpretable and patient-centered indicator of minimum disease burden (6, 7).

The characteristic of GMG is that about 80% of patients have acetylcholine receptor antibodies (AChR-Ab). The three main pathogenic mechanisms of AChR-Ab have been identified. The first type describes how AChR-Ab autoantibodies block acetylcholine from binding to the AChR, thereby hindering neuromuscular signal transduction (8). The second type is called antigen regulation, which describes the cross-linking of AChR-Ab, followed by receptor internalization, resulting in a reduction in the number of available receptors (9).The third type involves the activation of classical complement pathways by AChR-Ab (10). So, the complement system plays an important role in the pathogenesis of GMG.

Eculizumab is a monoclonal antibody that is administered intravenously and exerts its effects in systemic blood circulation by inhibiting the cleavage of C5, a key component of the complement cascade reaction (11). Tacrolimus (TAC), also called FK506, is a calcineurin inhibitor that decreases the transformation of B lymphocytes into plasma cells by influencing the activation and proliferation of T lymphocytes, which ultimately reduces the production of harmful antibodies (12). These two drugs have entirely different molecular mechanisms of action. Tacrolimus functions upstream in the molecular pathway of generalized myasthenia gravis (GMG), whereas Eculizumab acts downstream in this pathway (**Figure 1**). Both Tacrolimus and Eculizumab are currently widely used to treat GMG (13–15). However, there are no existing studies comparing the effectiveness and safety of using Eculizumab alone versus combining Eculizumab with Tacrolimus.

**Figure 1:**
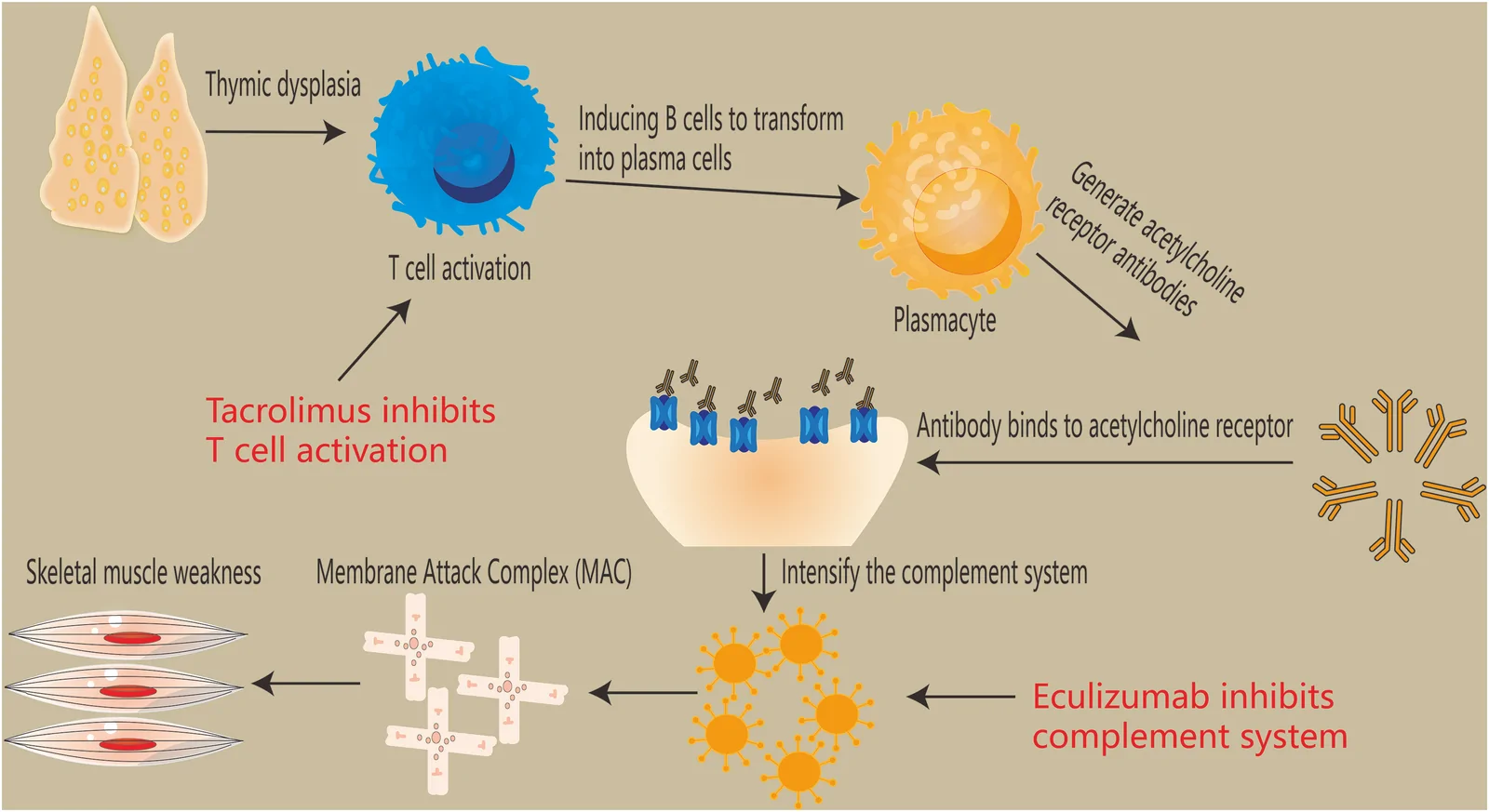
The mechanism diagram of tacrolimus and eculizumab in the treatment of myasthenia gravis is shown.

This study aims to compare the clinical effectiveness and safety of using Eculizumab alone versus a combination of Eculizumab and Tacrolimus in treating patients with GMG. Additionally, it seeks to further confirm the clinical efficacy and safety of Eculizumab in China, with the goal of providing effective treatment options for GMG patients.

## Materials and methods

### Study cohort

Our study includes a retrospective, observational, multicenter analysis involving patients from three university hospitals in Beijing, China (Peking University First Hospital, Beijing Hospital and Tsinghua University Yuquan Hospital This study has been approved by the local ethics committee of the coordination center (Approval No.: 2025BJYYEC-KY083-02) and conducted in accordance with the ethical standards set forth in the 1964 Helsinki Declaration and its subsequent amendments (16). We collected demographic and clinical data including age, gender, Myasthenia Gravis Foundation of America (MGFA) clinical classification (17), history of thymectomy, antibody status, CH50 concentration, Baseline (BL)doses of pyridostigmine and prednisolone, MG-ADL, QMG, Adverse events (AEs), occurrence of myasthenia gravis crisis, as well as disease progression and comorbidities. Projects related to MG are assessed by specialists in the MG domain.

### Patient selection

We identified 95 GMG patients treated with Eculizumab between September 2023 and December 2025. Among them, 33 patients were treated with both Tacrolimus and Eculizumab.

#### Inclusion criteria

1. Meet the international diagnostic criteria for generalized myasthenia gravis (17).
2. Age≥18 years old, starting treatment with Eculizumab and Eculizumab and Tacrolimus.
3. The detection of anti-AChR antibody was positive after the onset of myasthenia gravis.
4. Patients had available baseline assessments and a minimum follow-up of 6 months.
5. Combination therapy group patients must receive unchanged, full therapeutic doses of Tacrolimus for ≥12 weeks before screening.

#### Exclusion criteria

1. Patients who have received rituximab, immunoglobulin, or plasma exchange therapy within the 2 months prior to screening.

2. Patients who have used Tacrolimus within the 3 months prior to screening but discontinued it later.

6. Patients receiving other non-steroidal immunosuppressive therapies (NSIST).

7. All patients who have undergone thymectomy within the 6 months prior to screening, or have a clear pre-enrollment thymectomy plan at any time during the 26-week follow-up period.

8. Any patient with Tacrolimus initiation or dose modification within 12 weeks pre-screening was excluded.

3. Individuals with other autoimmune diseases (18).

### Treatment regimens

This retrospective cohort included patients treated with Eculizumab, with treatment regimens determined by routine clinical practice without randomization. Combination therapy (Eculizumab with Tacrolimus) was given to patients with AChR-positive gMG patients who had persistent symptoms after ≥12 weeks of fixed-dose Tacrolimus monotherapy. Tacrolimus was maintained to avoid disease flare from abrupt immunosuppressant withdrawal, while Eculizumab was added to inhibit complement-mediated neuromuscular damage. Only patients with unchanged Tacrolimus dosage for 12 weeks prior to Eculizumab initiation were included in the combination group.

Patients receiving Eculizumab monotherapy had no prior long-term Tacrolimus maintenance before complement inhibitor initiation, with individualized treatment decisions based on comprehensive clinical assessment of disease course and prior immunosuppressive response.

### Dosing regimen

Eculizumab is administered through load and maintenance phases. The loading phase includes intravenous infusion of 900mg of Eculizumab once a week, continuous intravenous infusion for 4 weeks, and intravenous infusion of 1200mg of Eculizumab once in week 5. Then, the patient receives intravenous infusion of 1200mg of Eculizumab every 2 weeks (11, 19–21). Tacrolimus administration regimen: 0.10mg/kg, twice a day (22), with a stable blood concentration of 5–8 ng/mL (23). Subsequently, adjust the dosage based on individual symptom responses to achieve optimal treatment outcomes (24–26).

### Evaluation metrics

#### Myasthenia gravis-activities of daily living

MG-ADL is an 8-item scale used to assess MG symptoms and their impact on daily activities (27). It evaluates the ability to engage in different activities in daily life, such as speaking, chewing, swallowing, breathing, brushing teeth or combing hair, standing up from a chair, as well as assessing diplopia and drooping eyelids. The scale is a discrete quantitative measure, with each of the 8 items scored from 0 to 3, resulting in a total score ranging from 0 to 24. Higher total scores indicate greater impairment. The MG-ADL assessment does not require any equipment to evaluate MG symptoms and their impact on daily functioning. The MG-ADL used in our research is an exclusively patient-reported outcome measure (PROM), with all questionnaires completed by patients on their own.

#### Quantitative myasthenia gravis

QMG quantifies disease severity based on physical function and structural damage, as defined by the International Classification of Functioning, Disability, and Health (28). The QMG consists of 13 items used to evaluate ocular, bulbar, and limb function. Among these, six are timed endurance tests measured in seconds. Each item is scored on a scale from 0 to 3, with a total possible score of 39. A higher total score indicates greater disease severity. The assessment is based on quantitative testing of specific muscle groups to evaluate limb function. It requires minimal equipment, including a spirometer, mouthpieces compatible with spirometers, nose clips, stopwatches, cups and water for swallowing tests, goniometers, and dynamometers, and is conducted through a physician’s examination.

### Assessment of CH50 concentration

CH50 concentration detection method (29, 30): (1) Solution sensitization: Prepare sensitized red blood cells by incubating 5% sheep red blood cells with an equal volume of hemolysin at 37 °C for 30 minutes. (2) Serum Gradient Dilution: Dilute the test serum with VBS buffer in a 1:1 ratio. Set up a complete lysis control and a red blood cell autolysis control. (3) Sample Incubation: Add the sensitized red blood cells to each tube and incubate in a 37 °C water bath for 60 minutes. (4) Centrifuge to measure absorbance: Centrifuge at 2000 rpm for 10 minutes, then measure the optical density (OD) of the supernatant at 540 nm. (5) Result Calculation: Calculate the serum dilution factor at 50% hemolysis, which corresponds to the CH50 value (U/mL).

### Definitions

The baseline was defined as the time of first administration of Eculizumab or Eculizumab and Tacrolimus. The analysis criteria of the respondents were that compared with the baseline, the MG-ADL decreased ≥ 2 points, and the QMG score decreased ≥ 3 points (31). Minimal symptom expression (MSE) is defined as MG-ADL score ≤1 (32).

### Study outcomes

The main outcome parameter is the proportion of GMG patients who have reached MSE status.

Secondary outcome measures: Changes in MG-ADL and QMG scores, as well as trends in the dosage of pyridostigmine and prednisolone, were analyzed. Considering the different treatment options under investigation, analyze using the best individual response for each case. For the analysis of PASS (Patient Acceptable Symptom Status), the following thresholds are used: MG-ADL score ≤ 2, QMG score ≤ 7 (33).

All data were collected during the BL period, 1 month (14–45 days after BL), 3 months (60–120 days after BL), and 6 months (150–210 days after BL). All participants completed the standardized 26 weeks core follow-up for the primary efficacy analysis. All 80 patients were followed up for 26 weeks, and 40 of them were extended to 52 weeks for long-term safety observation.

### Safety and adverse event assessment

Adverse events occurring during the 26-week follow-up period were systematically collected and documented. Routine laboratory assessments, including evaluations of hepatic and renal function, complete blood counts, and serum uric acid levels, were conducted to identify potential laboratory abnormalities. All adverse events—such as headache, rash, infections, pancreatitis, infusion-related reactions, and myasthenic crisis—were recorded, with their severity and potential association with the administered drugs thoroughly assessed. Multivariate logistic regression analysis with adjusted baseline characteristics was used to compare the incidence of adverse events between single drug therapy (Eculizumab) and combination therapy (Eculizumab and Tacrolimus). **Table 1** summarizes the results of all adverse events in our cohort.

**Table 1:** Summary of adverse events for all patients.

Adverse events	Eculizumab	Eculizumab and Tacrolimus	OR (95%CI)	P value
Any adverse event	13 (22.8)	9 (39.1)	0.576(0.186-1.780)	0.34
Myasthenic crisis	0(0)	0(0)	–	–
Infusion-related reaction event	1(1.8)	0(0)	–	0.99
Most common adverse events				
Headache	3(5.3)	1(4.3)	1.347(0.031-59.335)	0.88
Rash	1(1.8)	0(0)	0.960(0.067-13.714)	0.99
Urinary tract infection	1(1.7)	0(0)	–	0.99
Pancreatitis	1(1.7)	0(0)	–	0.99
Mild Upper respiratory tract infection	11(19.3)	6(26.1)	0.494	0.28
Renal impairment	3(5.2)	3(13.0)	0.454(0.073-2.830)	0.40
Liver dysfunction	2(3.5)	2(8.6)	0.011(0.000-15.181)	0.22
Hyperuricemia	2(3.5)	6(26.1)	0.094(0.014-0.634)	0.02

OR, Odds ratio; CI, 95% confidence interval; P values derived from multivariable logistic regression adjusted for baseline; Data are n (%).

### Statistical analysis

Perform statistical analysis using SPSS version 27.0 (IBM Corp) and visualize data using GraphPad Prism version 11.0 (GraphPad Software). Represent continuous variables with a normal distribution as mean ± standard deviation (SD), and use the independent samples t-test for intergroup comparisons, as appropriate. Represent non-normally distributed continuous data as median with interquartile range (IQR) and analyze using the Mann–Whitney U test or Wilcoxon signed-rank test. Categorical variables are presented as counts and percentages (n, %), and Fisher’s exact test is used for comparisons. 1:1 nearest-neighbor propensity score matching (PSM) was conducted to balance baseline confounding factors between the Eculizumab monotherapy group and combination group. Standardized mean difference (SMD) was computed for all covariates before and after matching to judge intergroup balance; covariates with absolute |SMD| ≥ 0.2 were defined as imbalanced, and absolute |SMD| < 0.1 was regarded as acceptable balance. For Myasthenia Gravis Foundation of America (MGFA) severity grades, Mann–Whitney U test was applied instead of SMD. All SMD data were summarized in **Supplementary Table S1**.

For continuous longitudinal indicators (QMG, ADL scale scores) measured at week 0, 4, 12 and 26, linear mixed models (LMM) with only subject random intercepts were adopted. Generalized linear mixed models (GLMM) with single random intercept were applied for longitudinal binary endpoints including MSE and clinical response rate. The variance inflation factor (VIF) of all fixed covariates was calculated to test multicollinearity, and all VIF values were less than 5, demonstrating no serious multicollinearity. Adjusted mean differences with 95% confidence intervals (CI) were reported for continuous outcomes, while adjusted risk differences (RD) and 95% CI were presented for binary longitudinal indicators at each follow-up time point.

Multivariable logistic regression was used to compare 26-week categorical outcomes (PASS status, adverse event incidence), and adjusted odds ratios (OR) together with corresponding 95% CI were reported. *Post hoc* pairwise comparisons were adjusted by the Bonferroni method. All partial follow-up missing data were processed by full information maximum likelihood (FIML) estimation built into GLMM, which assumes data missing at random (MAR). A p-value of less than 0.05 was considered statistically significant, and all tests were two-tailed.

## Results

95 patients were screened from September 5, 2023 to December 26, 2025. In the end, 80 patients were included in the study. The cohort of patients treated with Eculizumab comprised 57 patients. Another group treated with Eculizumab and Tacrolimus comprised 23 patients (**Figure 2**).

**Figure 2:**
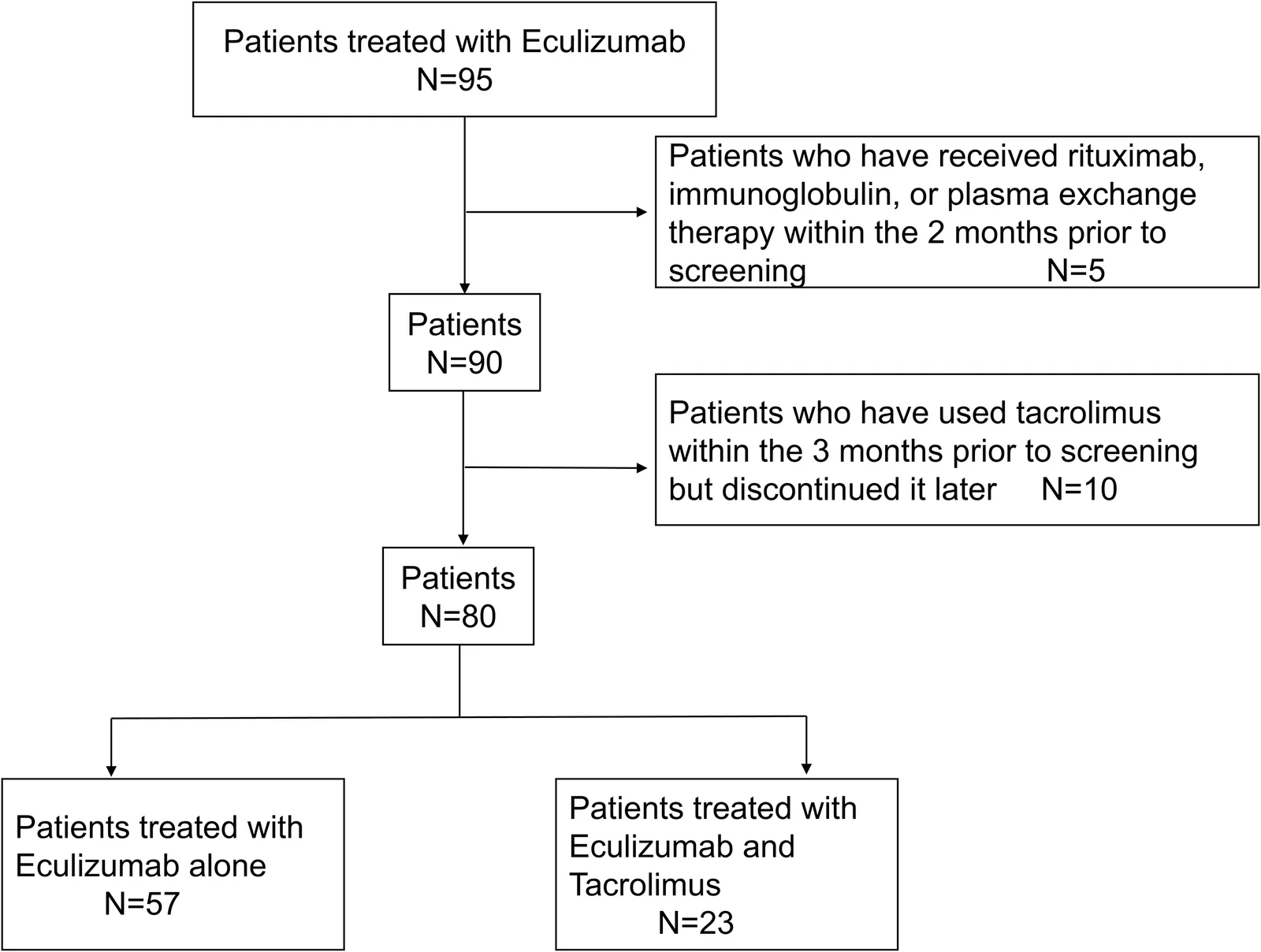
Flowchart illustrating participant selection for this study. Eighty patients fulfilled the inclusion and exclusion criteria and were assigned to two groups: 57 patients received Eculizumab only, while 23 patients were treated with a combination of Eculizumab and Tacrolimus.

The baseline characteristics of patients between two groups are presented in **Table 2**. According to the baseline variable SMD in **Supplementary Table S1**, age SMD>0.2. No significant overall difference in baseline MGFA distribution was found between two groups (Z = -0.725, P = 0.47), indicating balanced baseline disease severity. Fisher exact test showed that in patients with thymectomy history, there was no significant difference in thymoma resection rate between the Eculizumab treatment group (42.3%) and the Eculizumab and Tacrolimus treatment group (36.4%) (P>0.05). Finally, we included age, disease duration, baseline QMG and ADL scores, baseline pyridostigmine dosage and Pyridostigmine dosage as confounding variables in the fixed effects of GLMM.

**Table 2:** BL characteristics of patients treated with eculizumab (n=80).

Characteristics	Eculizumab(n=57)	Eculizumab and Tacrolimus(n=23)
Female patients, n (%)	32(56.1%)	14(60.9%)
Age	60(44.5,70.0)	66(59,71)
History of thymectomy, n (%)	26(45.6%)	11(47.8%)
Thymoma-induced thymectomy (%)	42.3%	36.4%
ADL score at BL, mean (SD)	10.91(5.48)	10.83(4.93)
QMG score at BL, mean (SD)	16.33(6.29)	16.83(5.58)
Prednisolone dose at BL, mg/day, mean (SD)	15.96(7.35)	16.68(7.23)
Pyridostigmine dose at BL, mg/day,mean (SD)	193.68(49.45)	200.87(58.92)
anti­AChR­Ab, n (%)	57(100)	23(100)
CH50 density at BL, U/mL, mean (SD)	50.88(9.7)	54.65(8.1)
Disease duration (year)	3(2, 5)	3(2, 7)
MGFA at BL, n (%)		
I	0(0)	0(0)
IIA	4(7.0)	5(21.7)
IIB	2(3.5)	3(13.5)
IIIA	11(19.3)	2(8.7)
IIIB	15(26.3)	4(17.4)
IVA	6(10.5)	1(4.3)
IVB	7(12.7)	2(8.7)
V	12(21.2)	6(26.1)

Ab, antibody; AChR, acetylcholine receptor; BL, baseline; MG­ADL, Myasthenia Gravis, Activities of Daily Living; MGFA, Myasthenia Gravis Foundation of America; QMG, Quantitaive Myasthenia Gravis; IQR, Interquartile Range; anti­AChR­Ab, n (%), denote the number and within-group percentage of AChR antibody-positive patients in each study group; Disease duration, Time since generalized myasthenia gravis diagnosis; Thymoma-induced thymectomy (%), Percentage refers to the proportion of thymectomy caused by thymoma in subjects with a history of thymectomy.

### Comparison of clinical efficacy between two groups

#### MG-ADL

Treatment with both therapies significantly reduced the MG-ADL score (p<0.001). But no significant group-by-time interactions were detected for MG-ADL, indicating a similar longitudinal trend between the two treatment groups (p=0.25, **Supplementary Table S3**). Regarding the MG­ADL, no relevant differences were observed at any time point (BL: Eculizumab 10.91 ± 5.48 vs Eculizumab and Tacrolimus 10.83 ± 4.93; month 1: 5.12 ± 4.46vs5.17 ± 3.85; month 3:3.30 ± 3.38vs 2.04 ± 2.29;p>0.05; **Figure 3A**).

**Figure 3:**
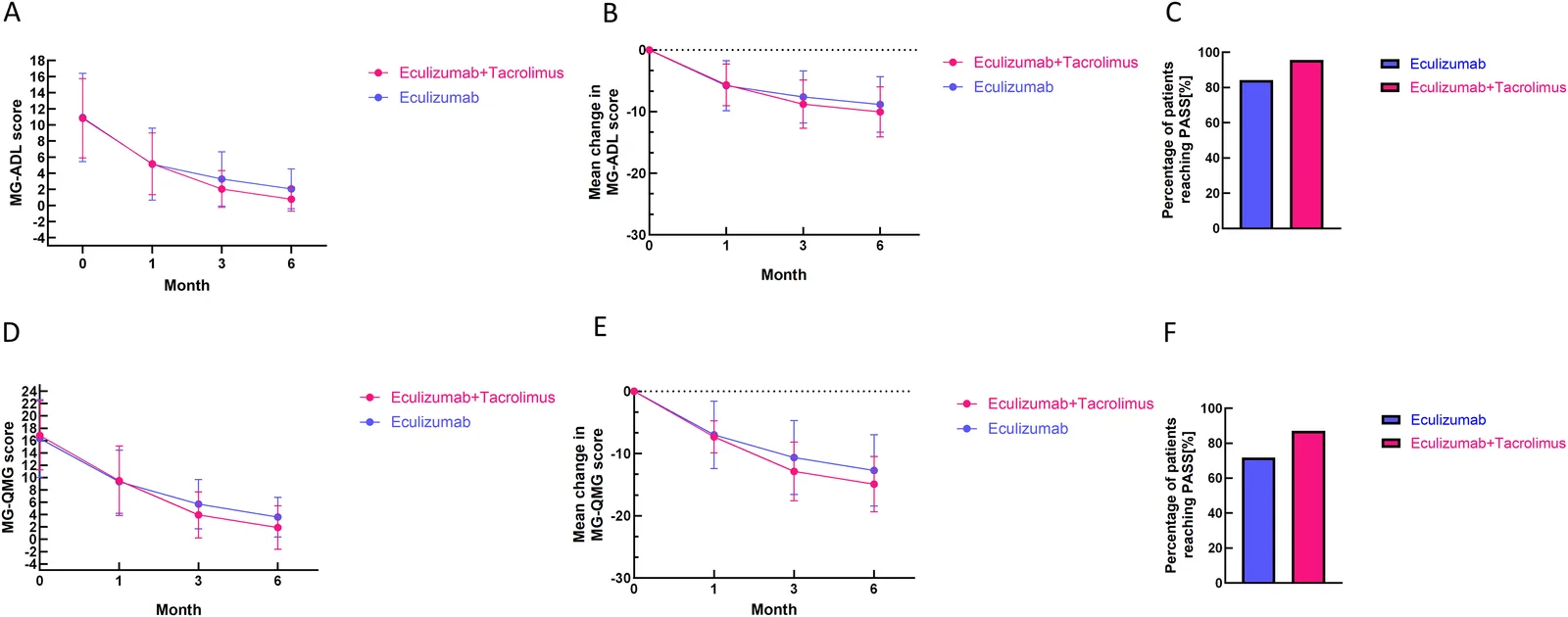
Comparison of clinical outcome parameters between patients treated with Eculizumab or Eculizumab and Tacrolimus. Individual changes in MG-ADL scores of patients treated with Eculizumab or Eculizumab and Tacrolimus at 1, 3, and 6 months after BL **(A)**; MG-ADL response in Eculizumab or Eculizumab and Tacrolimus-treated patients **(B)**; Percentage of patients who achieved PASS (ADL score ≤2) based on ADL score **(C)**;Individual changes in QMG scores of patients treated with Eculizumab or Eculizumab and Tacrolimus at 1, 3, and 6 months after BL **(D)**;QMG response in Eculizumab or Eculizumab and Tacrolimus-treated patients **(E)**; Percentage of patients who achieved PASS (QMG score ≤7) based on QMG score **(F)**;.

Compared to the baseline, the average difference between two groups at important time points gradually increases (**Figure 3B**, P<0.001). However, no significant difference was found for MG-ADL score reduction in the observed interval (interaction, p=0.16, **Supplementary Table S3**). A reduction of 8.84 ± 4.51 or 10.04 ± 4.05 points for MG­ADL score was found under Eculizumab or Eculizumab and Tacrolimus, respectively (p>0.05; **Figure 3B**).

Comparable numbers of patients in both groups met PASS criteria based on MG-ADL [Eculizumab 41(71.9%) vs Eculizumab and Tacrolimus 20(87%) patients; OR = 0.344(0.073-1.632), p>0.05, **Supplementary Table S4**; **Figure 3C**].

#### QMG

Treatment with both groups significantly reduced the QMG score (p<0.001) (**Figure 3D**). It gradually decreased with time (month 1, 3 and 6) and reached its lowest point in the sixth month. Concerning the QMG score, the Eculizumab group showed an average score of 16.33 ± 6.29 at BL, 9.33 ± 5.13 after 1 month, 5.70 ± 4.00 after 3 months and 3.61 ± 3.21 after 6 months (**Figure 3D**). In the Eculizumab and Tacrolimus group, the QMG scores were 16.83 ± 5.58 at BL,9.52 ± 5.64 after 1 month, 3.96 ± 3.75 after 3 months and 1.91 ± 3.52 after 6 months (**Figure 3D**). We did not find any significant differences when comparing Eculizumab vs Eculizumab and Tacrolimus-treated patients relative to the QMG score in different time (interaction, p=0.10, **Supplementary Table S3**).

Compared to the baseline, the average difference at investigating time points gradually increases (**Figure 3E**, P<0.001). We did not find any significant differences when comparing Eculizumab vs Eculizumab and Tacrolimus-treated patients relative to the QMG score reduction in the investigating time (**Figure 3E**, p>0.05). A reduction of 12.72 ± 5.74 or 14.91 ± 4.43 points in the QMG score was found under Eculizumab or Eculizumab and Tacrolimus, respectively (interaction, p=0.07, **Supplementary Table S3**; **Figure 3E**).

Comparable numbers of patients in both groups met PASS criteria based on QMG (Eculizumab 48(84.2%) vs Eculizumab and Tacrolimus 22(95.7%) patients; p>0.05) (OR =0.125 (0.008-1.964), p>0.05, **Supplementary Table S4**; **Figure 3F**).

#### Responder rate

The MG-ADL responder rate and the QMG responder rate gradually increases investigating time. Regarding MG-ADL responder rate, no relevant differences were observed at any time point (interaction, p=0.34, **Table 3**) (Eculizumab vs Eculizumab and Tacrolimus: month 1: 52(91.2%) vs 20(87%); month 3: 56(98.2%) vs 22(95.7%); month 6: 56(98.2%) vs 23(100%)) (**Figure 4A**). Concerning the QMG responder rate, no relevant differences were observed at any time point (interaction, p=0.41, **Table 3**) (month 1: 51(89.5%) vs 23(100%); month 3: 55(96.5%) vs 23(100%); month 6:56(98.2) vs 23(100%) (**Figure 4B**).

**Table 3:** Adjusted predicted non-response rates, proportion of patients without Minimal Symptom Expression (MSE) and intergroup differences by follow-up time.

Outcomes	Eculizumab	Eculizumab+ Tacrolimus	Difference in between two groups(Eculizumab- Eculizumab+ Tacrolimus)	95%CI	P
MG-ADL non-response rate					0.34†
0W	0.990	0.990	0.000	-1.624E-5to1.624E-5	0.99*
4W	0.088	0.130	-0.043	-0.152to0.066	0.44*
12W	0.022	0.045	-0.022	-0.067to0.022	0.33*
26W	0.022	0.010	-0.012	-0.014to0.038	0.38*
QMG non-response rate					0.41†
0W	0.999	0.999	0.000	-3.682E-6to3.667E-6	0.99*
4W	0.105	0.001	0.105	-0.009to0.219	0.07*
12W	0.035	0.001	0.035	-0.009to0.078	0.12*
26W	0.018	0.001	0.017	-0.007to0.041	0.17*
Proportion without MSE					0.006†
0W	0.971	0.971	0.000	-2.628E-5to2.627E-5	0.999*
4W	0.859	0.945	-0.085	-0.175to0.004	0.062*
12W	0.701	0.392	0.309	0.072to0.547	0.011*
26W	0.491	0.218	0.273	0.051to0.495	0.016*

CI, confidence interval; MG-ADL, myasthenia gravis daily living activity; QMG, Quantitaive Myasthenia Gravis; MSE, Minimal Symptom Expression; P* values are Bonferroni-corrected between-group comparison values at each visit; P† values for group-by-time interaction were based on fitting a generalized linear mixed model separately for each outcome variable.

The table presents predicted non-response probabilities from generalized linear mixed models. The main text describes treatment response; non-response probability is mathematically complementary to response probability. All raw data, model parameters and P values remain unchanged.

**Figure 4:**
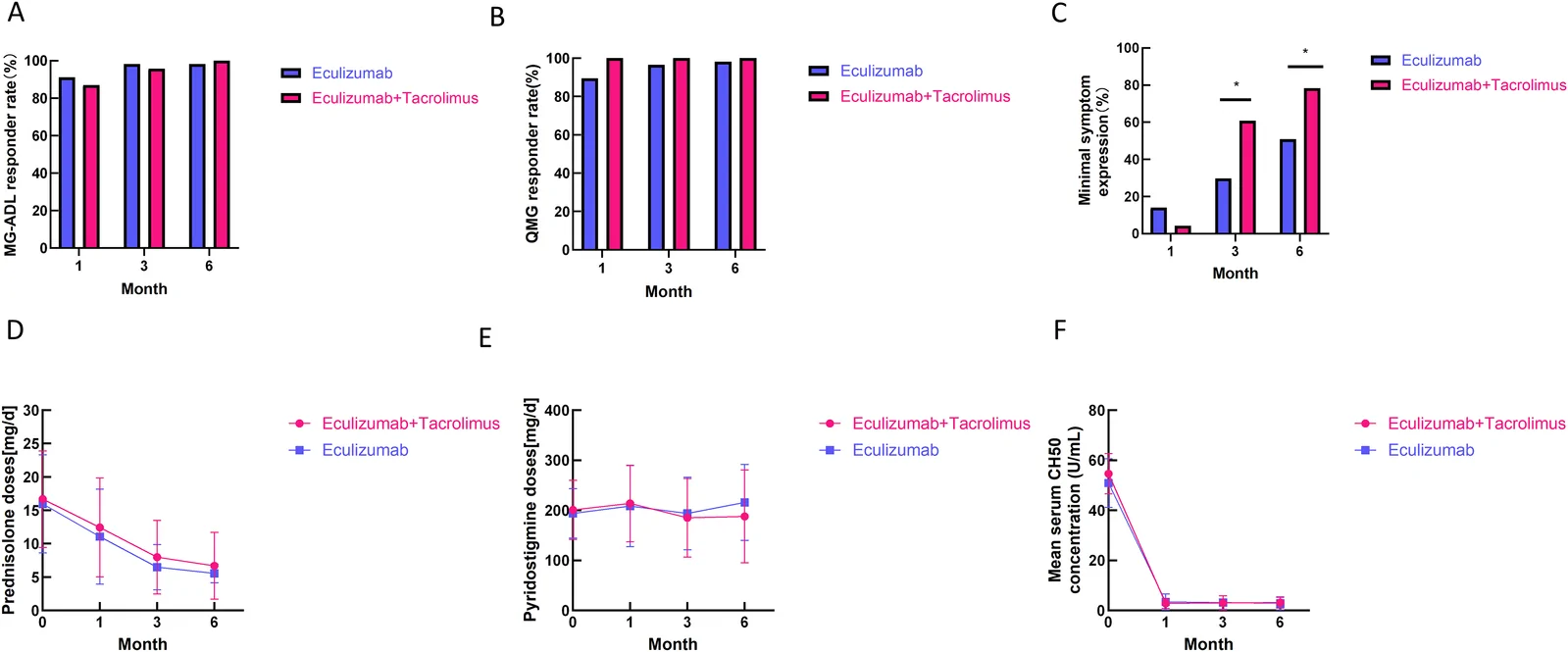
Comparison of clinical outcome parameters between patients treated with Eculizumab or Eculizumab and Tacrolimus. Responder Rate at the MG-ADL **(A)** and QMG scales **(B)**; Proportion of achieving MSE state **(C)**; The changes in dosage of Prednisolone **(D)** and Pyridostigmine **(E)**; CH50 concentration during treatment with Eculizumab and Eculizumab and Tacrolimus **(F)**. *p< 0.05.

#### MSE achievement rate

As the investigation time progresses, the proportion of GMG patients reaching MSE status gradually increases (**Figure 4C**). Generalized linear mixed models revealed a significant group-by-time interaction for the proportion of patients achieving MSE (P = 0.0006, **Table 3**), indicating that the longitudinal trend of MSE attainment differed significantly between the Eculizumab monotherapy group and the Eculizumab plus Tacrolimus combination group. Regarding the proportion of achieving MSE state at different time periods, no relevant differences were observed in the first month (Eculizumab 8(14%) vs Eculizumab and Tacrolimus 1(4.3%) (P>0.05, **Figure 4C**). The difference between the two groups is significant in the third month (Eculizumab 17(29.8%) vs Eculizumab and Tacrolimus 14(60.9%); p<0.05) and remained significant in the sixth month (Eculizumab 29(50.9%) vs Eculizumab and Tacrolimus 18(78.3%); p<0.05) (**Figure 4C**).

#### Prednisolone dose reduction

Baseline Prednisolone dose was 15.96± 7.35 mg/day for Eculizumab-treated patients and 16.68±7.23 mg/day for Eculizumab and Tacrolimus-treated patients (p>0.05). At the end of the follow-up period, mean dose was 5.57 ± 1.42 for Eculizumab and 6.68 ± 4.99 mg/day for Eculizumab and Tacrolimus (interaction, p=0.92, **Supplementary Table S3**; **Figure 4D**). There is no significant difference in the therapeutic effect between the two groups.

#### Pyridostigmine dose reduction

Baseline Pyridostigmine dose was 193.68 ± 49.45 mg/day for Eculizumab-treated patients and 200.87± 58.92 mg/day for Eculizumab and Tacrolimus-treated patients (p>0.05). At the end of the follow-up period, mean dose was 215.79 ± 75.95 for Eculizumab and 187.83± 92.78 mg/day for Eculizumab and Tacrolimus (p>0.05) (**Figure 4E**). There is no significant difference in the therapeutic effect between the two groups (interaction, p=0.48, **Supplementary Table S3**).

#### Pharmacodynamics

Over time, the concentration of CH50 rapidly decreased to its lowest point after complement inhibition therapy and remained at a stable level (**Figure 4F**). This Indicated that the treatment of the both groups had an inhibitory effect on the complement pathway. Nevertheless, there was no significant difference in the changes of complement concentration between the two groups (interaction, p=0.23, **Supplementary Table S3**).

### Safety

During the whole follow-up period, none of our patients received remedial treatment, including IVIG or plasma exchange for MG exacerbation. During the study period, there were no deaths among the 57 patients in the Eculizumab group and the 23 patients in the Eculizumab and Tacrolimus group. 13 out of 57 patients (22.8%) in the Eculizumab group and 9 out of 23 patients (39.1%) in the Eculizumab and Tacrolimus group had adverse events, with the most common being headaches, rash, urinary tract infections, pancreatitis, upper respiratory tract infections, renal dysfunction, liver dysfunction, and hyperuricemia (**Table 1**).

Incidence of Hyperuricemia was significant difference between groups (Eculizumab 2(3.5%) vs Eculizumab and Tacrolimus 6(26.1%) patients; OR = 0.094(0.014-0.634), p<0.05); The proportion of severe crisis and infusion related reaction events is 0%. There was no significant difference in the incidence of other events such as headache, rash, urinary system infection, pancreatitis, upper respiratory tract infection, renal dysfunction, and liver dysfunction between the two groups.

## Discussion

Our study is the first real-world report comparing the efficacy of Eculizumab alone versus the combination of Eculizumab and Tacrolimus in GMG. Patients were treated with either Eculizumab alone or the combination therapy according to clinical practice at multiple hospitals in Beijing, China. We compared and analyzed the efficacy and safety of these treatment approaches. Our findings indicate that both groups experienced similar clinical benefits, including reduced disease burden, improved physical strength, enhanced quality of life, and overall benefit to GMG patients. Furthermore, we observed a correlative link between Eculizumab and Tacrolimus combination treatment and an increased probability of achieving MSE status, in comparison with Eculizumab monotherapy. However, combination therapy is associated with a higher risk of elevated serum uric acid among GMG patients, which necessitates regular close monitoring of renal function.

The absolute decrease in MG-ADL score is not significantly different from the previously reported use of Eculizumab in four Italian GMG reference centers (–8.84vs–7.00), but far exceeds that in the REGAIN trial (-4.2) (11, 15). The reason for this situation may be due to differences in baseline characteristics such as race and weight, which make domestic myasthenia gravis patients more sensitive to treatment with Eculizumab (34, 35). Previous studies have reported that the absolute decrease in MG-ADL scores of myasthenia gravis patients treated with tacrolimus alone was approximately 6 scores (36). However, the efficacy of combination of Eculizumab and Tacrolimus in patients with GMG has not been reported domestically or internationally. In our research, the absolute decrease in MG-ADL scores was higher than previously reported for tacrolimus alone for the treatment of myasthenia gravis(-10.04vs-6.00). Compared with using tacrolimus alone, the combination of Eculizumab and Tacrolimus may be more effective in improving the quality of daily life of patients with myasthenia gravis. However, there was no significant difference in the decrease in MG-ADL scores between using Eculizumab alone and in combination with Eculizumab and tacrolimus(-8.84vs-10.04). There is no significant difference in improving the quality of life of GMG patients between the two treatment methods.

Both groups showed significant improvement in QMG scores after 1 month of treatment. After 3 and 6 months of treatment, the improvement of QMG in patients treated with both treatments was relatively smooth. The absolute improvement in patients receiving treatment with Eculizumab was higher than previously reported in the REGAIN trial (-12.72vs-4.7) or in the previously reported the study of four Italian GMG reference centers. The reason for this situation may still be related to differences in race or significant weight differences between domestic and foreign countries. In the study, there was no significant difference in the decrease in MG-ADL scores between using Eculizumab alone and in combination with Eculizumab and Tacrolimus (-12.70vs-14.91). Due to the fact that the QMG scale can objectively reflect the muscle strength of GMG patients (28, 37–40), there is no significant difference in improving muscle strength between Eculizumab alone and in combination with Eculizumab and Tacrolimus in patients.

According to the MG-ADL/QMG scale, the response rate of GMG patients to the treatment of both groups gradually increases with the change of treatment time (**Figures 4A, B**). In the sixth month of treatment, the response rate of both groups of GMG patients reached over 95%. Although we found no difference in response rate analysis between the two groups, this study indicates that AChR Ab+ GMG patients have high sensitivity to both groups of drug therapy.

When comparing our results with the REGAIN trial, we analyzed MGFA Class V and thymoma-related baseline features. MGFA Class V patients accounted for 21.2% of the Eculizumab monotherapy group and 26.1% of the Eculizumab plus Tacrolimus group, while the rates of prior thymectomy were 45.6% and 47.8% in the two arms. Nonparametric tests confirmed balanced baseline disease severity and thymic disease history between our two groups. However, the overall proportion of patients with MGFA Class V crisis and thymoma-associated myasthenia gravis (TAMG) in our cohort was significantly different from that of the REGAIN trial (11, 21). Previous studies have validated that MGFA Class V respiratory crisis and TAMG blunt the early therapeutic response to Eculizumab (41). Such inter-trial disparities in TAMG prevalence leads to heterogeneous clinical outcomes, making direct cross-study efficacy comparisons inappropriate. Nevertheless, recent real-world data further complements this perspective: thymoma associated MG (TAMG) patients experience faster early symptom relief with Eculizumab than non-TAMG patients, with equivalent long-term clinical outcomes (42). Even so, sustained disease control remains challenging for TAMG patients, who typically require combination therapy with corticosteroids, conventional immunosuppressants, and Eculizumab (41, 43, 44). This conclusion is consistent with our observational results, which demonstrate that combined therapy with Eculizumab and Tacrolimus can realize effective long-term symptom control in patients with GMG and prior thymic abnormalities.

The proportion of patients who achieved MSE was higher than previously reported Eculizumab in clinical practice in adults with MG in the United States (50.9%vs33%) (45). This may be due to the smaller sample size of our study compared to the previous one. In the first month of treatment, there was no significant difference in the proportion of patients who achieved MSE (MG­AD ≤ 1 point) between the two groups (Eculizumab (14.0%) vs Eculizumab and Tacrolimus (4.3%)). However, there was a significant difference in the proportion of patients who reached MSE status between the two groups during the third and sixth months of treatment. We observed a correlative link between Eculizumab and Tacrolimus combination treatment and an increased probability of achieving MSE status, in comparison with eculizumab monotherapy. This study provides a good treatment plan for GMG patients.

Reducing prednisolone is a key feature of innovative therapies, and the combination of Eculizumab and the combination of Eculizumab and Tacrolimus can significantly reduce baseline doses. Our study found an Eculizumab steroid-sparing effect of –65.10% of baseline dose, which is similar to the previously reported value of-79.12% despite a different baseline mean dose (15). A research report suggests that tacrolimus has a prednisolone-sparing effect in patients with myasthenia gravis (46). However, in a systematic review and meta-analysis study, it was found that compared to the placebo group, Tacrolimus did not show a significant difference in its prednisolone saving effect (47). In our study, we found that there was no significant difference in the savings of prednisolone between the combined use of Eculizumab and Tacrolimus compared to using Eculizumab alone. This study suggests that Tacrolimus may not have a significant effect in saving prednisolone during the treatment of GMG patients.

CH50 is a functional assay that provides a quantitative measurement of the overall activity of classical complement pathway (48). In this study, we found that both groups can significantly reduce the concentration of complement CH50 in the blood of GMG patients. The decrease in complement CH50 concentration is accompanied by a decrease in QMG and MG-ADL scores, indicating that Eculizumab treats GMG through the mechanism of complement system, which is consistent with previous research findings (48–50).

The current methods for treating myasthenia gravis have both short-term and long-term side effects, which may limit their clinical application. In this study, both treatment groups showed good tolerability, with most adverse events being mild or moderate in severity and a lower incidence of infusion reactions. Although headache is the most common adverse event, there was no significant difference in the incidence of headache between the two treatment groups. The infection rate is of particular concern as patients with GMG are prone to infection, and immunosuppressive therapy may exacerbate the infection (51, 52). The infection rate between the two groups is very low and there is no significant difference (**Table 1**). The infection between the two groups was mild to moderate, and no severe infection was found. Previous studies have shown that Tacrolimus can cause hyperuricemia in patients, mainly because Tacrolimus inhibits renal uric acid excretion and increases uric acid levels (53). Eculizumab can effectively control mg symptoms, prolong the administration time of tacrolimus, reduce inflammatory state, and synergistically weaken the renal uric acid secretion ability (21). In our study, we observed consistent results that compared to the use of Eculizumab alone, the combination of Eculizumab and Tacrolimus treatment in GMG patients resulted in elevated uric acid levels (OR = 0.094(0.014-0.634), p<0.05). However, GMG patients with high uric acid levels did not show any symptoms. In clinical practice, for patients taking tacrolimus, clinical doctors should pay attention to the adverse reactions of this drug and closely monitor their uric acid levels.

The results of our follow-up of patients with GMG in China using Eculizumab are basically consistent with the results of the multi center real cohort in the United States and the national observational study in Japan (45, 54). Comparable declining trends in MG-ADL and QMG scores were observed across all studied populations. However, subtle ethnic variations were noted: Chinese patients with MG tend to achieve a stable minimal symptom state more rapidly when treated with a combination of Eculizumab and Tacrolimus. Additionally, the incidence of hyperuricemia among Chinese patients receiving Tacrolimus is relatively elevated, a phenomenon infrequently reported in Western cohorts. These regional disparities underscore the importance of individualized monitoring strategies for Asian MG patients undergoing combined immunosuppressive therapy.

This study has several limitations. Firstly, the limitations of this study lie in the small total number of patients which may lead to bias. Further expansion of sample size and prospective randomized cohort studies with multiple centers are needed in the future. Secondly, a significant limitation of this study is that the follow-up time is relatively short, which may limit the conclusion of long-term sustained efficacy and delayed adverse events. Thirdly, minor weight-based individual dose differences inevitably exist within this real-world cohort. Fourthly, in the baseline distribution of MGFA grade between the two groups, there was a slight visual difference in the proportion of MGFA IIa subtypes, although statistical analysis confirmed that there was no significant difference between the groups, which should be taken into account when interpreting our results. Finally, retrospective data collection is prone to information bias such as missing or inconsistent medical records. Accordingly, we cannot draw definitive causal conclusions regarding the superiority of combination therapy over monotherapy. only associative relationships can be inferred from the current data.

## Conclusion

Overall, our real-world research indicates that both Eculizumab and Tacrolimus alone provide significant clinical benefits in reducing symptoms and improving quality of life, with both therapies demonstrating superior efficacy and safety. we observed a correlative link between Eculizumab and Tacrolimus combination treatment and an increased probability of achieving MSE status. To further validate these findings, future prospective randomized cohort studies with large sample sizes and multiple centers are needed.

## Data Availability

The original contributions presented in the study are included in the article/**Supplementary Material**. Further inquiries can be directed to the corresponding authors.
