# Supplementary material for: Clinical study of eculizumab with or with not tacrolimus in the treatment of generalized myasthenia gravis
Source: Front Immunol. 2026 Oct 7;17:1906981. doi: 10.3389/fimmu.2026.1906981 (PMC13683607; doi:10.3389/fimmu.2026.1906981)
Supplement: Supplementary file 1 [file Table1.docx]

**Supplementary Table S1 BL characteristics of patients treated with Eculizumab**

| Characteristics | Eculizumab(n=57) | Eculizumab and Tacrolimus(n=23) | Standardized Mean Difference | P |
| --- | --- | --- | --- | --- |
| Female patients, n (%) | 32（56.1%） | 14（60.9%） | 0.095 | 0.70 |
| Age | 60(44.5,70.0) | 66（59,71） | 0.423 | 0.17 |
| History of thymectomy, n (%) | 26(45.6%) | 11(47.8%) | 0.043 | 0.86 |
| ADL score at BL, mean (SD) | 10.91(5.48) | 10.83(4.93) | -0.017 | 0.99 |
| QMG score at BL, mean (SD) | 16.33(6.29) | 16.83(5.58) | 0.088 | 0.84 |
| Prednisolone dose at BL,  mg/day, mean (SD) | 15.96(7.35) | 16.68(7.23) | 0.100 | 0.69 |
| Pyridostigmine dose at BL, mg/day,mean (SD) | 193.68(49.45) | 200.87(58.92) | 0.122 | 0.58 |
| anti­AChR­Ab, n (%) | 57(100) | 23(100) | - | - |
| Disease duration (year) | 3(2，5) | 3(2，7) | 0.088 | 0.92 |
| MGFA at BL, n (%) |  |  |  | 0.47 |
| I | 0(0) | 0(0) | - | - |
| IIA | 4(7.0) | 5(21.7) | - | - |
| IIB | 2(3.5) | 3(13.5) | - | - |
| IIIA | 11(19.3) | 2(8.7） | - | - |
| IIIB | 15(26.3) | 4(17.4) | - | - |
| IVA | 6(10.5) | 1(4.3) | - | - |
| IVB | 7(12.7) | 2(8.7) | - | - |
| V | 12(21.2) | 6(26.1) | - | - |
| Ab, antibody; AChR, acetylcholine receptor; BL, baseline; MG­ADL, Myasthenia Gravis-Activities of Daily Living; MGFA, Myasthenia Gravis Foundation of America; QMG, Quantitative Myasthenia Gravis; IQR, Interquartile Range. anti­AChR­Ab, n (%), denote the number and within-group percentage of AChR antibody-positive patients in each study group; Disease duration, Time since generalized myasthenia gravis diagnosis; | | | | |

**Supplementary Table S2 Diagnostic results for all generalized linear mixed models (GLMM)**

| Diagnostic item | Test content & results | Conclusion |
| --- | --- | --- |
| Multicollinearity test (all fixed covariates) | Variance inflation factor (VIF) of all confounding covariates (age, disease duration, baseline QMG and ADL scores, baseline pyridostigmine dosage and glucocorticoid dosage) were calculated; all VIF values < 5 | No severe multicollinearity detected |
| Random effect structure setting | Only subject-level random intercept was specified, without random slope of time; covariance structure = Variance Component (VC) | Simplified random structure to reduce convergence failure risk |
| Random intercept variance (primary binary response model) | Estimated variance of random intercept =0.321 | Variance > 0, parameters identifiable, no singular matrix |
| Model convergence warning check | No warning of "Hessian matrix not positive definite" generated during SPSS model fitting | All GLMM models converged stably with valid parameter estimation |

**Supplementary Table S3 Longitudinal adjusted mean values of continuous clinical indicators and intergroup comparisons at each follow-up time point.**

|  | Eculizumab | | Eculizumab+ Tacrolimus | | Between-group difference | | |
| --- | --- | --- | --- | --- | --- | --- | --- |
|  | Mean (SD) 95% CI | | Mean (SD) 95% CI | | 95% CI F P | | |
| MG-ADL scores 0.25† | | | | | | | |
| BL | 10.883(0.352) | 10.190to11.576 | 10.899(0.556) | 9.804to11.995 | -1.318to1.286 | 0.001 | 0.98 |
| W4 | 5.093(0.352) | 4.401to5.786 | 5.247(0.556) | 4.151to6.343 | -1.456to1.148 | 0.054 | 0.82 |
| W12 | 3.269(0.352) | 2.576to3.961 | 2.117(0.556) | 1.021to3.212 | -0.150to2.454 | 3.041 | 0.08 |
| W26 | 2.041(0.352) | 1.348to2.733 | 0.856(0.556) | -0.240to1.951 | -0.117to2.487 | 3.217 | 0.07 |
| Mean change in MG-ADL score 0.16† | | | | | | | |
| BL | -0.004(0.548) | -1.093to1.084 | 0.010(0.864) | -1.707to1.727 | -2.052to2.023 | 0.000 | 0.99 |
| W4 | -5.794(0.398) | -6.583to-5.004 | -5.642(0.630) | -6.890to-4.394 | -1.634to1.330 | 0.041 | 0.84 |
| W12 | -7.618(0.293) | -8.199to-7.038 | -8.772(0.464) | -9.693to-7.851 | 0.058to2.251 | 4.356 | 0.04 |
| W26 | -8.846(0.295) | -9.436to-8.256 | -10.033(0.467) | -10.969to-9.097 | 0.072to2.301 | 4.548 | 0.04 |
| QMG scores 0.10† | | | | | | | |
| BL | 16.407(0.462) | 15.496to17.317 | 16.645(0.731) | 15.205to18.084 | -1.949to1.473 | 0.075 | 0.78 |
| W4 | 9.407(0.462) | 8.496to10.317 | 9.340(0.731) | 7.901to10.780 | -1.644to1.777 | 0.006 | 0.94 |
| W12 | 5.775(0.462) | 4.865to6.685 | 3.775(0.731) | 2.335to5.215 | 0.289to3.711 | 5.307 | 0.02 |
| W26 | 3.687(0.462) | 2.777to4.597 | 1.731(0.731) | 0.292to3.171 | 0.245to3.667 | 5.075 | 0.02 |
| Mean change in QMG score 0.07† | | | | | | | |
| BL | -0.096(0.730) | -1.547to1.355 | 0.237(1.153) | -2.052to2.527 | -3.050to2.384 | 0.059 | 0.81 |
| W4 | -7.096(0.568) | -8.222to-5.970 | -7.067(0.897) | -8.847to-5.287 | -2.143to2.085 | 0.001 | 0.98 |
| W12 | -10.727(0.412) | -11.545to-9.910 | -12.632(0.654) | -13.929to-11.336 | 0.361to3.449 | 5.983 | 0.02 |
| W26 | -12.815(0.384) | -13.580to-12.050 | -14.676(0.610) | -15.891to-13.461 | 0.413to3.308 | 6.563 | 0.01 |
| Prednisolone dose 0.92† | | | | | | | |
| BL | 16.070(0.546) | 14.994to17.145 | 16.425(0.863) | 14.724to18.126 | -2.376to1.665 | 0.120 | 0.73 |
| W4 | 11.157(0.546) | 10.082to12.233 | 12.186(0.863) | 10.485to13.887 | -3.049to0.992 | 1.006 | 0.32 |
| W12 | 6.596(0.546) | 5.520to7.672 | 7.730(0.863) | 6.029to9.430 | -3.154to0.887 | 1.221 | 0.27 |
| W26 | 5.675(0.546) | 4.599to6.751 | 6.425(0.863) | 4.724to8.126 | -2.771to1.270 | 0.535 | 0.47 |

**Continued Supplementary Table S3 Longitudinal adjusted mean values of continuous clinical indicators and intergroup comparisons at each follow-up time point**

| Eculizumab Eculizumab+ Tacrolimus Between-group difference | | | | | | | | | |
| --- | --- | --- | --- | --- | --- | --- | --- | --- | --- |
| Mean (SD) 95% CI | | | Mean (SD) 95% CI | | | 95% CI F P | | | |
| Pyridostigmine dose 0.48† | | | | | | | | | |
| BL | 193.533(9.350) | 175.131to211.936 | | 201.243(14.766) | 172.181to230.305 | | -42.213  to26.793 | 0.193 | 0.66 |
| W4 | 208.270(9.350) | 189.868to226.673 | | 214.287(14.766) | 185.225to243.349 | | -40.519  to28.486 | 0.118 | 0.73 |
|  |  |  | |  |  | |  |  |  |
| W12 | 193.533(9.350) | 175.131to211.936 | | 185.591(14.766) | 156.529to214.653 | | -26.561  to42.445 | 0.205 | 0.65 |
| W26 | 215.639(9.350) | 197.236to234.041 | | 188.200(14.766) | 159.138to217.262 | | -7.064  to61.942 | 2.450 | 0.12 |
| CH50 concentration 0.23† | | | | | | | | | |
| BL | 50.846(1.164) | 48.530to53.161 | | 54.745(1.833) | 51.098to58.392 | | -8.222to0.423 | 3.221 | 0.08 |
| W4 | 3.393(0.375) | 2.648to4.139 | | 2.959(0.954) | 1.778to4.139 | | -0.969to1.838 | 0.379 | 0.54 |
| W12 | 3.099(0.350) | 2.404to3.795 | | 3.120(0.555) | 2.019to4.222 | | -1.331to1.289 | 0.001 | 0.98 |
| W26 | 2.884(0.396) | 2.092to3.676 | | 3.287(0.626) | 2.033to4.540 | | -1.892to1.087 | 0.293 | 0.59 |
| SD, standard deviation; BL, baseline; CI, confidence interval; MG-ADL, myasthenia gravis daily living activity; QMG, Quantitaive Myasthenia Gravis; F values represent test statistics for between-group comparison at each visit, adjusted by Bonferroni correction; P values are Bonferroni-corrected between-group comparison values at each visit; P† values for group-by-time interaction were based on fitting a linear mixed model separately for each outcome variable. | | | | | | | | | |

**Supplementary Table S4 Proportion of patients achieving PASS status at week 26 and intergroup comparisons using adjusted logistic regression.**

|  | Eculizumab（n=57） | Eculizumab+ Tacrolimus(n=23) | OR (95%CI) | P |
| --- | --- | --- | --- | --- |
| PASS(ADL) | 41(71.9%) | 20(87%) | 0.344(0.073-1.632) | 0.179 |
| PASS(QMG) | 48(84.2%) | 22(95.7%) | 0.125(0.008-1.964) | 0.139 |
| OR, Odds ratio; CI, 95% confidence interval; Patient Acceptable Symptom Status (PASS); MG-ADL, myasthenia gravis daily living activity; QMG, Quantitaive Myasthenia Gravis; P values were derived from multivariable logistic regression adjusted for baseline confounding variables to compare the difference in PASS attainment rates between groups. | | | | |
